# The Roles of Secreted Wnt Ligands in Cancer

**DOI:** 10.3390/ijms24065349

**Published:** 2023-03-10

**Authors:** Johannes Werner, Kim E. Boonekamp, Tianzuo Zhan, Michael Boutros

**Affiliations:** 1 Division of Signaling and Functional Genomics, German Cancer Research Center (DKFZ), Medical Faculty Mannheim, Heidelberg University, 69120 Heidelberg, Germany; 2 Medical Faculty Heidelberg, Heidelberg University, 69120 Heidelberg, Germany; 3 Department of Medicine II, Medical Faculty Mannheim, Mannheim University Hospital, Heidelberg University, D-68167 Mannheim, Germany; tianzuo.zhan@umm.de; 4 Mannheim Cancer Center, Medical Faculty Mannheim, Heidelberg University, D-68167 Mannheim, Germany; 5 DKFZ-Hector Cancer Institute, University Medical Center Mannheim, D-68167 Mannheim, Germany

**Keywords:** Wnt signaling, Wnt ligands, cancer, non-canonical Wnt signaling, tumor microenvironment, metastasis, immunotherapy, targeted therapy

## Abstract

Wnt ligands are secreted signaling proteins that display a wide range of biological effects. They play key roles in stimulating Wnt signaling pathways to facilitate processes such as tissue homeostasis and regeneration. Dysregulation of Wnt signaling is a hallmark of many cancers and genetic alterations in various Wnt signaling components, which result in ligand-independent or ligand-dependent hyperactivation of the pathway that have been identified. Recently, research is focusing on the impact of Wnt signaling on the interaction between tumor cells and their micro-environment. This Wnt-mediated crosstalk can act either in a tumor promoting or suppressing fashion. In this review, we comprehensively outline the function of Wnt ligands in different tumor entities and their impact on key phenotypes, including cancer stemness, drug resistance, metastasis, and immune evasion. Lastly, we elaborate approaches to target Wnt ligands in cancer therapy.

## 1. Introduction

In 1973, the first genomic locus of a Wnt ligand (wingless) was discovered as a crucial component for wing formation in Drosophila melanogaster [1]. In the following years, several other loci whose disruption caused segmentation defects during Drosophila development, a phenotype linked to Wnt signaling, were found to encode genes evolutionarily conserved in many other species, including vertebrates [2]. Wnt ligands (WNTs) were linked to cancer when the insertion of the mouse mammary tumor virus (MMTV) into the promoter of int1, the murine ortholog of wingless (later termed Wnt1), was discovered to induce mammary tumors in mouse models [3]. Later, it was shown in hereditary colorectal cancers that hyperactivation of the Wnt pathway is closely linked to carcinogenesis in humans [4,5,6,7]. These findings have initiated many research efforts to uncover the function of components of the Wnt pathway and its physiological roles, including WNTs, in health and disease. In this review, we provide an overview of the various roles WNTs play in tumorigenesis and tumor progression.

### 1.1. Secretion of WNTs

The family of WNTs comprises 19 distinct genes in humans [8]. WNTs are cysteine-rich proteins of approximately 40 kDa size [9], which are secreted via the endoplasmic reticulum (ER) and the Golgi apparatus by a machinery of proteins assisting post translational modification and transport through the secretory pathway [8]. Upon their translocation into the ER lumen, glycosaminoglycans are attached to the nascent WNTs at different sites [10,11]. Additionally, the ER-resident acyltransferase Porcupine attaches palmitoleic acid to specific cysteine and serine residues of the WNTs [12]. Subsequently, the cargo protein Wntless/evenness (WLS/Evi) binds to WNTs by binding to the palmitoleic acid and guides them through the secretory pathway [13,14,15,16,17]. Upon secretion from the producing cell, modified WNTs are presented to the recipient cells, either bound to carrier proteins, such as afamin, or on exosomes or cytonemes to exert their effects in an auto- or paracrine manner [8,18]. The cargoreceptor Evi/Wls is recycled from the plasma to the ER by a retromer-dependent mechanism to be loaded again with WNTs [19,20]. Recently, it has been shown that the abundance of WLS/Evi in the ER is regulated by its ubiquitination and subsequent degradation, thereby restricting the secretion of WNTs (Wolf and Boutros, Development, in press) [21].

### 1.2. The Role of Secreted WNTs in Canonical and Non-Canonical Wnt Signaling

Wnt signaling can be categorized into canonical, β-catenin-dependent signaling, and several non-canonical, β-catenin-independent pathways. In development and homeostasis, canonical Wnt signaling is induced by binding of WNTs to their cognate receptors on the cell membrane. WNTs bind to Frizzled (FZD) receptors and Lrp5/6 co-receptors, resulting in Disheveled (DVL) polymerization and subsequent inactivation of the destruction complex. DVL polymerization leads via additional steps to the accumulation and nuclear translocation of β-catenin. In the nucleus, β-catenin binds to members of the TCF/LEF transcription factor family and subsequent expression (or inhibition) of Wnt target genes, contributing to cell differentiation, proliferation, or maintenance of stemness [22,23]. In the absence of WNTs, the destruction complex, a large protein multimer comprising the scaffolding proteins APC and AXIN1/2, the serin/threonin kinases CK1 and GSK-3β, and the E3 ubiquitin ligase β-TrCP, targets the transcriptional co-activator β-catenin for proteasomal degradation [22,24]. Canonical Wnt signaling can be augmented by R-spondin ligands, which increase the abundance of FZDs on the plasma membrane by binding to LGR5 receptors and consequently inhibiting the E3 ubiquitin ligases RNF43/ZNRF3, which target FZDs for degradation [25,26].

Unlike canonical Wnt signaling, which drives the expression of transcriptional targets, non-canonical Wnt signaling comprises different pathways that do not result in the stabilization of β-catenin. The Wnt/planar cell polarity (PCP) and the Wnt/Ca^2+^ pathways are both activated by specific WNTs, such as WNT5A and WNT11, but result in the activation of distinct signaling cascades [27]. In the Wnt/PCP pathway, non-canonical WNTs bind to FZD receptors and co-receptors, such as VANGL, ROR1/2, and PTK7, which leads to cellular and transcriptional responses, including activation of the Rho/ROCK and JNK signaling cascades. As one consequence, alterations of the cell morphology and motility are induced [28,29,30,31]. In Wnt/Ca^2+^ signaling, binding of WNTs to FZD receptors results in a swift increase in intracellular Ca^2+^ levels and subsequent activation of Ca^2+^-dependent proteins. For instance, Wnt/Ca^2+^ signaling can directly influence cell function independent of transcription by activating protein kinase C (PKC) or calmodulin-dependent protein kinases (CAMK). Additionally, increased Ca^2+^ levels lead to transcriptional changes by modulating the activity of NFAT and NF-κB [32,33].

Another β-Catenin-independent pathway, Wnt/STOP signaling, requires components of the canonical Wnt cascade upstream of β-Catenin, such as LRP6 receptors and DVL [34,35,36]. Wnt/STOP signaling results in the reduction of the GKS3-dependent polyubiquitination of a variety of proteins and, therefore, increases their abundance. An important target of Wnt/STOP is the cell cycle regulator c-MYC [34]. Among the effects mediated by Wnt/STOP signaling are the regulation of cell size and correct microtubule assembly during mitosis. Recently, it has been discovered that most WNTs fail to activate Wnt/STOP signaling. WNT10B was identified as an essential ligand for activation of Wnt/STOP signaling in human cancer and somatic cell lines [35]. The relevance of this novel Wnt pathway in cancer biology remains to be elucidated, but recent findings indicate an involvement of Wnt/STOP in ribosome biogenesis in human pancreatic cancer models [37].

WNTs can be classified into activators of canonical or non-canonical Wnt pathways. Initial studies in *Xenopus* showed that Xwnt1, -3A, -8, and -8b activate canonical Wnt signaling, whereas it was shown that non-canonical WNTs can inhibit canonical Wnt signaling, either via competition at the receptor level or by inducing caspase-mediated cleavage of β-catenin [38,39,40]. Furthermore, Xwnt5A, -4, and -11 can exert additional effects [41,42]. In Table 1, the effects of each WNT ligand on Wnt signaling and other oncogenic signaling pathways observed in human cancers are summarized.

### 1.3. The Expression and Regulation of WNT Ligands in Cancer

**Figure 1:**
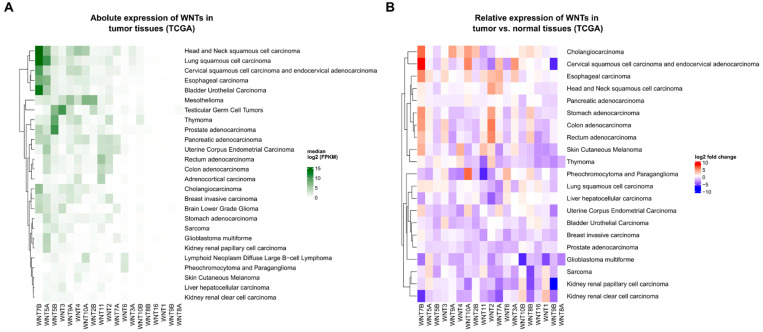
**Expression of WNT ligands in different tumor entities.** (**A**) Transcript abundance of all WNT genes in a panel of selected cancer entities. High expression is indicated in green. WNTs are specifically expressed across different cancer entities, e.g., WNT7B is highly expressed in cancers derived from squamous cell epithelium. For all available datasets of tumor tissues, log2 of the median fragments per kilobase million (FPKM) values of all samples is shown. (**B**) Relative expression of all WNT genes in a panel of cancer entities compared to corresponding normal tissues. Upregulation of a gene in cancer tissues is shown in red, while downregulation is indicated in blue. (**A**,**B**) Data were obtained from the TCGA database using the *TCGAbiolinks* package in R [98]. Heatmaps were generated using the *Complexheatmap* package [99]. For all datasets providing normal tissue gene expression data, median FPKM values were separately calculated for normal and tumor tissues. From these values, log2(fold change) was calculated.

Most research on Wnt signaling in cancer has focused on the biological consequences of mutations of Wnt pathway components, such as APC or β-catenin. However, WNTs are also differentially expressed in various cancer entities, such as breast cancer and colorectal cancer (CRC), as shown in an analysis of RNA sequencing data from the TCGA database (Figure 1) [47,52,79,80]. As each specific WNT ligand plays distinct roles in development and tissue homeostasis, their expression needs to be tightly controlled on a spatio-temporal level [81]. The expression of WNT genes is regulated by a variety of (tissue-specific) transcription factors and finetuned post-transcriptionally by non-coding RNAs [82,83]. In carcinogenesis, tumor cells often hijack these regulatory mechanisms to increase the expression of specific WNTs. For instance, the upregulation of *WNT11* observed in small cell lung cancer cell lines was found to be facilitated by the oncogenic transcription factor ASCL1 [76]. Another example is that the transcription factor and known tumor suppressor GATA4 inhibits *WNT7B* expression via TGF-β signaling in murine lung cancer models [84]. Similarly, GATA6, a tumor-suppressive transcription factor that is mutationally inactivated in pancreatic cancer, suppresses the expression of WNT7B [85,86]. In t(1;19) pre-B acute lymphoblastoid leukemia (ALL), the oncogenic fusion protein E2a-pbx1 drives the expression of *WNT16* [87]. Moreover, specific cases of post-transcriptional control of WNT transcripts by miRNAs or lncRNAs have also been described [46,65,68,72,88,89,90,91,92,93,94,95]. For instance, the expression of *WNT1* was shown to be regulated by miRNA-148a in non-small cell lung cancer [90], and miRNA-200b in gastric cancer and CRC [46,94]. Overall, a dysregulated expression of various WNTs in diverse cancer types was described, but the functional consequences has remained elusive in most cases.

Notably, not only WNTs are implied in the regulation of WNT signaling in tumor cells and their microenvironment. In multiple myeloma, for instance, Dickkopf-1 (DKK1), an inhibitor of WNT-receptor-interaction, secreted by myeloma cells takes a key role in formation of osteolytic lesions by inhibiting osteoblast differentiation [96,97].

## 2. The Interplay of Secreted WNTs and Oncogenic Signaling Pathways

**Figure 2:**
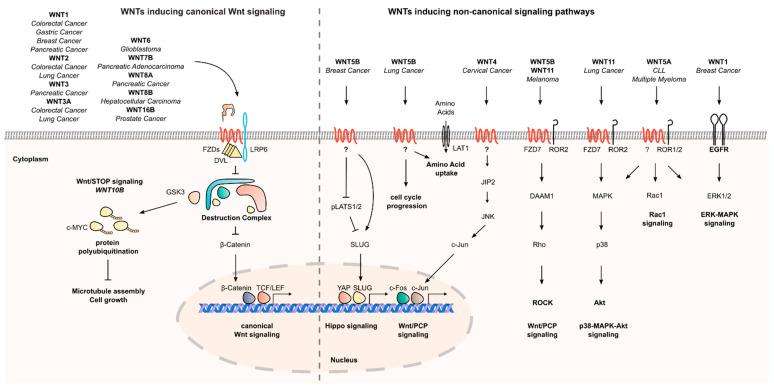
**Overview of mechanisms by which secreted Wnt ligands promote cancer cell proliferation.** Several WNTs, among those WNT1, WNT2, and WNT3A, activate canonical Wnt signaling to promote proliferation. Beside effects on canonical Wnt signaling, WNTs act on multiple other pathways to stimulate cell growth. For instance, WNT5B promotes cell cycle progression and uptake of amino acids in lung cancer, as well as stimulates Hippo signaling in breast cancer. WNT4, WNT5B, and WNT11 activate Wnt/PCP signaling. WNT5B and WNT11 furthermore activate p38-MAPK signaling in melanoma, while WNT1 activates ERK1/2 signaling in an EGFR-dependent manner. WNT10B has been shown to activate GSK3-dependent Wnt/STOP signaling.

WNT proteins have pleiotropic effects during homeostasis and development, partly due to modulation of signaling cascades resulting in altered gene transcription, Ca^2+^ levels and cytoskeletal rearrangements. Thus, WNTs can contribute to a multitude of oncogenic processes. Even though different WNTs can exhibit overlapping effects on signaling pathways, there are many examples of context- and tissue-specific effects of individual WNTs. Here, we describe the involvement of specific WNTs in β-catenin-dependent and -independent signaling cascades, which contribute to cancer initiation and progression (Figure 2).

### 2.1. Oncogenic Effects of Secreted WNTs

Hyperactivation of canonical Wnt/β-catenin signaling is often observed during carcinogenesis. Many in vitro and in vivo studies show that in different tumor types, such as colorectal, lung, breast, or hepatocellular cancer, specific WNTs, including WNT1, WNT2, WNT3A, WNT6, WNT8B, WNT7B, and WNT16B, can activate canonical Wnt signaling and support tumor proliferation [43,46,47,48,50,67,69,71,78,92,94,100]. Interestingly, knockdown of *WNT3* and *WNT3A* reduces Wnt reporter activity in CRC cell lines with mutant *APC*, indicating that WNTs can stimulate canonical Wnt signaling even in the presence of Wnt pathway mutations [52].

Beyond their impact on canonical Wnt signaling, many tumor-promoting effects of specific WNTs were linked to their effects on β-catenin-independent pathways. For example, Rodriguez-Hernandez et al. reported an important role of Wnt/PCP signaling for melanoma progression, which was induced by WNT5B and WNT11 [73]. In melanoma cell lines, knockdown of *WNT5B* and *WNT11* reduced the formation of melanospheres. Knockdown of *FZD7* and *DAAM1*, as well as pharmacological inhibition of Rho-associated protein kinase (ROCK), could reproduce this effect, and furthermore, reduce tumor volume in a xenograft mouse model of melanoma. Additionally, a reduction in the invasiveness of melanoma cells in a collagen invasion assay upon knockdown of *WNT5B* and *WNT11* was observed. Mechanistically, it was shown that WNT11 controls proliferation and invasion by binding to FZD7 receptors and downstream activation of the Rho-ROCK1/2-Myosin II signaling pathway [73]. Another interaction of WNTs and components of the PCP pathway was demonstrated in cervical cancer cell lines and xenograft mouse models. Here, WNT4 was shown to be a mediator of oncogenic effects of the viral oncoprotein E6. Upon silencing of *E6*, a reduced translation of *WNT4* and JNK interacting protein 2 (*JIP2*) could be observed by polysome profiling. Overexpression of either *WNT4* or *JIP2* could rescue the proliferative defect upon E6 silencing. This effect correlated with phosphorylation of JNK, suggesting a link between Wnt/PCP and JNK signaling in this model [101]. Further, the Wnt/Ca^2+^ pathway was reported to sustain tumor proliferation. In prostate cancer cell lines, knockdown of *WNT7B* reduced cell proliferation. This effect could be reproduced by knockdown of different PKC isoenzymes, which are central mediators of Wnt/Ca^2+^ signaling, as well as by the knockdown of the PKC substrate *MARCKS*. Upon ectopic expression of *WNT7B*, an increased phosphorylation of MARCKS could be observed, while knockdown of *WNT7B* conferred the opposite effect. This supports the hypothesis that WNT7B enhances prostate cancer cell proliferation of by activating PKC-MARCKS signaling [70]. Furthermore, an interplay between WNTs with other signaling pathways besides Wnt/PCP and Wnt/Ca^2+^ signaling was described by several studies.

For instance, in lung cancer cell lines and transgenic mouse models, WNT7B was shown to be the downstream effector of a GATA4–TGFB2 signaling axis which mediates cell senescence. Knockdown of *WNT7B* in lung cancer cell lines could phenocopy the senescence-inducing effect of ectopic GATA4 expression, while *WNT7B* overexpression could rescue this effect. Interestingly, the effects of WNT7B were not mediated by canonical Wnt signaling, as knockdown or pharmacological inhibition of β-catenin could not reproduce the pro-senescent effect of *WNT7B* abrogation [84]. In another study in lung cancer cell lines, knockdown of *WNT5B* reduced colony formation ability. Mechanistically, knockdown of *WNT5B* induced cell cycle arrest and altered the expression of cell cycle-associated proteins. Additionally, WNT5B was shown to induce the expression of the amino acid transporter *LAT1*. Overexpression of *LAT1* could partly rescue the growth defect conferred by knockdown of *WNT5B*, underlining its important role in mediating the biological effects of WNT5B [65].

In breast cancer cells, depletion of *WNT5B* by RNAi decreased the formation of mammospheres, which could be rescued by overexpression of TAZ, indicating an interplay between Wnt and Hippo signaling on breast cancer initiation [64]. In small cell lung cancer (SCLC), WNT11 has been shown to act on cell proliferation and p38/AKT signaling. Upon knockdown of WNT11, the growth of two SCLC cell lines was reduced. Conversely, overexpression of *WNT11* enhanced cell growth and was associated with increased phosphorylation of p38 and AKT [76]. Another interplay of a WNT with a MAPK signaling cascade was demonstrated in a mouse breast cancer model, where WNT1 activates an EGFR–ERK1/2 signaling axis, which contributes to tumor cell proliferation [43]

Oncogenic effects of secreted WNTs do not remain confined to solid tumor entities. WNT5A signaling, for instance, has been reported to promote proliferation in several hematological cancers. In multiple myeloma, WNT5A mediates the adhesion of myeloma cells to bone marrow cells via a ROR2 and AKT-dependent mechanism [102]. In chronic lymphocytic leukemia (CLL), WNT5A was shown to be secreted from nurse-like cells (NLC) in the CLL microenvironment in the bone marrow and secondary lymphoid tissues. In CLL cells, NLC-derived WNT5A was shown to enhance proliferation in a ROR1-dependent manner [103]. Furthermore, treatment of CLL cells with WNT5A in vitro led to phosphorylation of ERK1/2 and increased cell proliferation [63]. Altogether, WNT5A has been shown to activate non-canonical pathways through ROR1/2 receptors in hematological malignancies.

### 2.2. Tumor-Suppressive Effects of Secreted WNTs

While many tumor-promoting effects of secreted WNTs have been described, some WNTs also display tumor-suppressive effects. Contradictory to other studies showing an involvement of WNT5A in CRC progression [104,105], the promoter of *WNT5A* was found to be frequently methylated in CRC tissues and cell lines, but not in normal colon tissue [106]. Supporting a tumor suppressive function of WNT5A, two studies showed that its overexpression in the CRC cell line HCT116 could reduce Wnt reporter activity, β-catenin protein abundance and tumor growth in mouse xenograft experiments [106,107]. Ambiguous roles have also been described for WNT7A in gastric cancer. The *WNT7A* promoter was shown to be frequently methylated in gastric cancer, leading to its transcriptional downregulation. Overexpression of *WNT7A* in gastric cancer cells decreased the expression of the EMT markers Snail and Vimentin. Furthermore, reduced cell invasion, migration, and proliferation of gastric cancer cells were observed upon overexpression of *WNT7A*. In mouse xenograft experiments, overexpression of *WNT7A* reduced the tumor volume [108]. However, Wang et al. showed that WNT7A is upregulated in gastric cancer tissues compared to normal gastric mucosa and that depletion of *WNT7A* by RNA interference reduces migration and invasion capabilities of gastric cancer cells in transwell and Matrigel invasion assays [89]. Furthermore, the canonical WNT3 is upregulated in CLL cells compared to normal B-cells, but a relatively low expression of WNT3 is associated with a poor prognosis [109]. In B-ALL primary cells and cell lines, treatment with WNT3A activated expression and nuclear translocation of β-catenin and reduced leukemia proliferation [110]. These results indicate a possible tumor-suppressive effect of canonical WNTs in lymphocytic leukemia.

To summarize, specific WNTs show diverse tumor-promoting, but also tumor-suppressive effects in different cancer models. These phenotypes can mainly be attributed to the modulation of canonical Wnt signaling, but due to their pleiotropic effects, WNTs also promote cell proliferation by influencing other oncogenic signaling cascades.

## 3. Secreted WNTs in Cancer Stemness

In homeostasis, the self-renewal of stem cells is often controlled by niches containing WNT-secreting cells [111,112]. In intestinal crypts, for instance, this niche consists of stromal and Paneth cells, while in hair follicles, a niche of self-sustaining quiescent stem cells with autocrine WNT secretion exists [111,112,113]. A similar principle can be observed in several cancer entities, in which subsets of cells, so-called cancer stem cells (CSC), are indispensable for tumor maintenance and progression [114]. Wnt signals can be critical for maintaining cancer stemness, as several studies show. For instance, Tammela et al. demonstrate that the spheroid-forming ability of lung cancer cells was drastically increased by treatment with recombinant WNT3A, while pharmacological inhibition of endogenous WNT secretion had the opposite effect. Using a lung cancer mouse model, they also showed that pharmacological inhibition or genetic depletion of *Porcn* or *Lgr5* reduces tumor formation. In this murine model, a subset of WNT-secreting tumor cells was identified, which constitutes a niche sustaining proliferative signaling and tumor progression [115].

In Wnt-dependent cancers characterized by mutations in *RNF43/ZNRF3* or by *RSPO*-fusions, WNTs provided by the tumor cells or the microenvironment are essential for tumor propagation [116,117]. For instance, in a mouse model of WNT-dependent CRC with intestine-specific mutations of *Rnf43* and *Znrf3*, a Wnt3 secreting niche provided by Paneth cells was necessary to maintain tumor growth [118]. Another study underlines the importance of a WNT-dependent CSC population for tumor progression. In a human patient-derived xenograft mouse model harboring *PTPRK*–*RSPO3* fusions, which amplify canonical Wnt signaling driven by WNTs, treatment with anti-RSPO antibodies led to reduced tumor volumes, loss of stem cell properties, and induction of differentiation [119]. Following this logic, by losing their dependence on growth-factor-secreting niches, cancer cells can increase their malignant potential [114]. This niche independence can be achieved through mutations in Wnt pathway components downstream of the receptor level or by upregulation of autocrine WNT secretion. For example, Seino et al. demonstrated that WNT-dependent human pancreatic cancer organoids can be classified into two subtypes, of which one depended on stromal WNTs, while the other exhibited autocrine secretion of WNTs [85]. It was shown that pancreatic organoids with engineered oncogenic mutations in *KRAS*, *CDKN2A*, *TP53*, and *SMAD4* can acquire independence of a stromal WNT-secreting niche by upregulating endogenous *WNT7B* expression [85]. A similar result was shown in a mouse model of chemically induced colon cancer without mutations in core Wnt pathway components (AOM/DSS). Here, the expression of *Wnt7a/Wnt7b* was upregulated in tumor cells compared to a healthy colon. In this model, blockage of WNT secretion from tumor cells by conditional knockout of *Wls*/*Gpr177* increased the proportion of apoptotic and differentiated tumor cells, highlighting the importance of endogenous WNT secretion to maintain self-renewing capacity [120]. Furthermore, WNTs derived from the tumor microenvironment can contribute to cancer cell stemness, and co-culture of ovarian cancer stem cells with M2-polarized macrophages increased the expression of the stem cell marker *ALDH* in the cancer cells, an effect which could be reversed by blockade of WNT secretion, as well as knockdown of *WNT5B* in macrophages [121].

The examples described above show that WNT secretion from tumor or stroma cells promote carcinogenesis by inducing stem cell like phenotypes. However, a well-balanced secretion of WNTs can also protect against cancer initiation. In a VillinCre^ER^APC^fl/fl^ mouse model, pharmacological inhibition of Wnt secretion favors growth of *APC* mutant cells. The proposed mechanism is that *APC* mutant cells do not require WNTs for growth, thereby outcompeting untransformed colon epithelial cells, which depend on WNTs secreted in the crypt [122]. Furthermore, two studies showed that in human colon organoid models, *APC* mutant cells secrete inhibitors of canonical Wnt signaling, e.g., NOTUM or WIF1, to gain a growth advantage over WNT-dependent wild type stem cells [123,124]. These results demonstrate the importance a well-regulated WNT secreting niche, not only for epithelial homeostasis, but also for prevention of tumor initiation in colon.

Taken together, the discussed studies highlight the different roles of WNTs derived from the tumor and adjacent stroma cells for cancer stemness and therapy resistance. WNTs can, depending on the presence of cancer mutations, sustain cancer cell stemness, but also restrict cancer initiation indirectly by supporting the growth and differentiation of adjacent healthy cells

### Secreted WNTs and Drug Resistance

Cancer treatment traditionally relies on three pillars: surgery, radiotherapy, and chemotherapy [125]. In the last decades, targeted and immunomodulatory therapies were added to this repertoire [125,126,127,128]. A challenge to cancer therapy is that cancer cells often acquire therapy resistance during the course of treatment. In this context, CSCs play an important role, as they were frequently found to be intrinsically resistant to chemo- and radiotherapy [129,130], an observation that was mechanistically linked to hyperactive Wnt signaling [131,132,133].

In line with these observations, several WNTs were functionally linked to chemo- and radiotherapy resistance. For instance, WNT7B and FZD7 have been found to be overexpressed on both the RNA and protein level in gemcitabine-resistant pancreatic cancer cell lines. Upon treatment with gemcitabine in these cell lines, knockdown of *WNT7B* or *FZD7* reduced the proportion of cells expressing the CSC markers CD44 and CD24, as well as increasing the proportion of apoptotic cells and reducing expression of β-catenin [134]. Additionally, siRNA mediated knockdown of *WNT2B* in ovarian cancer cell lines treated with paclitaxel or cisplatin, and increased cell death and apoptosis [135,136]. Furthermore, *WNT6* expression in gastric cancer tissues was negatively correlated with response to chemotherapy. In line with these results, knockdown of *WNT6* reduced survival in gastric cancer cells treated with anthracyclines [137]. Moreover, WNT8A has been linked to radiation resistance. Clofibrate was shown pancreatic cancer cells sensitize to radiation. Mechanistically, it could then be shown that clofibrate abrogates *WNT8A* expression and consequentially reduces canonical Wnt activity [138].

Besides radio- and chemotherapy, WNTs modulate the response to targeted agents. In melanoma patients undergoing treatment with BRAF inhibitors (BRAFi), *WNT5A* expression in tumor tissues correlates with therapy response [59]. Upon long-term treatment with BRAFi, melanoma cell lines upregulate *WNT5A* expression. Knockdown of *WNT5A* by RNAi in BRAFi-resistant cell lines results in a lower activity of Akt signaling and reduced viability upon BRAFi treatment. The effects of WNT5A in these models were shown to depend on FZD7 and RYK receptors, emphasizing the role of non-canonical Wnt signaling in this process [139]. Another study showed that WNT5A stabilized p53 in *TP53* wild type melanoma cell lines and thereby renders the cells resistant to BRAFi. Consequently, inhibition of p53 induced by WNT5A sensitized the melanoma cells to treatment with BRAF and MEK inhibitors [57].

Not only tumor-intrinsic WNT secretion is implicated in therapy resistance, but also ligands provided by the tumor microenvironment. In prostate cancer, for instance, genotoxic stress induced by radio- and chemotherapy induces the expression of *WNT16B* in cancer-associated fibroblasts (CAF) in vitro and in tumor biopsies. Furthermore, it was shown that knockdown of *WNT16B* by shRNA in fibroblasts reduced prostate cancer cell viability after treatment with mitoxantrone [78]. Similarly, WNT3A could be detected in conditioned medium derived from CRC-associated fibroblasts. Addition of this medium to CRC cell lines increased the volume of xenograft tumors generated from these cells upon treatment with 5-fluorouracil and oxaliplatin. Concomitantly, an increased canonical Wnt activity and expression of the stem cell markers Nanog and CD133 was observed in these tumors. In vitro, addition of exogenous WNT3A to CRC spheres during treatment with oxaliplatin increased sphere number, while blockade of WNT secretion by porcupine inhibition diminished it, indicating a vital effect of WNT3A on oxaliplatin resistance of CRC cells [140]. Two different studies showed that in CLL, WNT5A has been reported to contribute to resistance to the BTK inhibitor Ibrutinib [61] and the BCL-2 inhibitor Venetoclax [62], in both cases via a ROR1-dependent signaling axis [61,62]. In mouse xenograft models of CLL, treatment with the anti-ROR1-antibody Cirmtuzumab further reduced spleen volume, which indicates a suppressive effect on CLL progression [61].

In summary, WNTs secreted both from tumors and adjacent stroma cells can contribute to chemo- and radiotherapy resistance by enhancing canonical Wnt signaling and inducing cancer cell stemness.

## 4. Secreted WNTs and Metastasis

**Figure 3:**
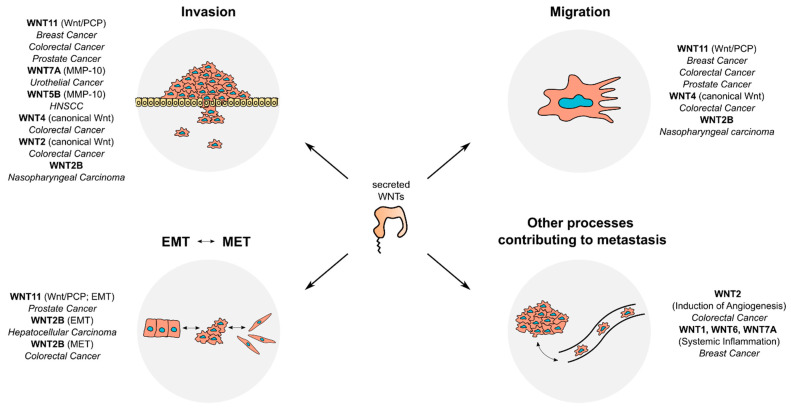
**Mechanisms by which WNTs support metastasis.** WNTs contribute to key processes of metastasis, including tumor cell invasion and migration. Furthermore, WNT11 and WNT2B contribute to the transition between epithelial and mesenchymal states of cancer cells. EMT, epithelial to mesenchymal transition; MET, mesenchymal to epithelial transition.

Cancer development is a multistep process that involves tumor initiation, tumor progression, and metastasis. Microscopically, the process of metastasis can be subdivided into different subprocesses, namely dissemination and invasion, intravasation, circulation, extravasation, and colonization [141,142]. In epithelial cancers, EMT is one of the hallmarks of metastasis. During EMT, tumor cells gain a more mesenchymal phenotype, which is characterized by altered expression of specific markers, such as a reduction of E-cadherin levels and an increase of vimentin and N-cadherin [143]. Furthermore, matrix-metalloproteinases (MMPs) play important roles for metastasis by cleaving extracellular matrix and thereby facilitating tumor cell invasion [144]. In this section, we highlight the roles of WNTs in subprocesses of metastasis (Figure 3).

### 4.1. Canonical WNTs and Metastasis

Beyond initiation of carcinogenesis, hyperactivation of canonical Wnt signaling can also modulate advanced tumor phenotypes, such as metastasis. In a study in breast cancer, a canonical Wnt signaling signature was shown to be enriched in triple-negative breast cancer (TNBC), a subtype that exhibits high invasiveness and metastatic potential. In TNBC-derived cell lines, either knockdown of β-catenin or pharmacological inhibition of WNT secretion could diminish invasion and migration abilities in in vitro assays [145]. The involvement of canonical WNTs in breast cancer metastasis is further supported by findings from a transgenic mouse model. Here, ablation of *Trp53* induced the expression of multiple WNTs, including *Wnt1*, *Wnt6*, and *Wnt7a*, which coincided with an increased Wnt signaling gene expression signature. Those WNTs in turn locally induced the secretion of IL-1β in tumor-associated macrophages, consequently promoting systemic inflammation and facilitating pulmonary metastasis. Interestingly, this phenotype could be reverted by inhibition of WNT secretion with a porcupine inhibitor [146].

Pro-metastatic effects of canonical Wnt signaling were shown to depend on WNTs in other cancers, as well. For instance, WNT2 secreted from CAFs was shown to activate canonical Wnt signaling in CRC cell lines and to promote CRC cell invasion and induce angiogenesis in 3D co-culture assays [49,50]. Another study showed that in the CRC cell lines HCT116 and HT29, hypoxia induced the secretion of exosomes that contain a high amount of WNT4. Those exosomes subsequently stimulated the invasion and migration abilities of these cell lines in vitro, which could be reverted by knockdown of *WNT4* or pharmacological inhibition of β-catenin signaling [55]. In a subclone of the urothelial bladder carcinoma (UBC) cell line 5637, which was selected for its increased invasion capability, *WNT7A* was shown to be overexpressed compared to the parental cell line, in parallel to an increased level of active β-catenin. In concordance with these results, treatment of UBC cell lines with recombinant WNT7A induced the invasive behavior in transwell assays. Consistently, tail vein injection of UBC cells transfected with a *WNT7A* expression plasmid in mice resulted in an increase of lung metastasis foci. Mechanistically, it could be shown that WNT7A induced the expression of several EMT markers, such as Vimentin and of Matrix-Metalloprotease 10 (MMP10). In this case, the expression of MMP10 was demonstrated to be regulated by two TCF/LEF binding sites, suggesting an important role of canonical Wnt signaling [68]. A similar link between WNT5B, cell invasion, and the expression of *MMP10* was also observed in squamous head and neck squamous cancer cell lines [66]. For WNT2B, another ligand known to activate canonical Wnt signaling, multifaceted roles in metastasis were reported. For instance, it was shown that knockdown of *WNT2B* reduces invasion and migration in in vitro assays in nasopharyngeal carcinoma cell lines [95]. Furthermore, WNT2B-conditioned medium was shown to induce the expression of EMT markers in hepatocellular carcinoma (HCC) cells, while medium of cells with knockdown of *WNT2B* conferred the opposite effect [51]. Conversely, in an in vitro model of EMT in CRC, WNT2B was shown to confer the opposite effect, as it has been shown to be critical for the mesenchymal to epithelial transition (MET) of the CRC cell line LIM1863 [147]. These results show that WNTs can influence the transition between epithelial and mesenchymal states of cancer cells in both directions.

### 4.2. Non-Canonical WNTs and Metastasis

The non-canonical Wnt/PCP and Wnt/Ca^2+^ signaling pathways are strongly implied in metastatic processes, as they are able to influence cytoskeletal remodeling, cellular motility, and migration [29,148]. Particularly, the non-canonical ligands WNT5A and WNT11 were shown to contribute to metastasis in several cancer entities. For instance, in breast cancer, WNT11 was shown to bind the non-canonical receptor ROR2. Increased *ROR2* expression is inversely associated with metastasis-free survival, and *ROR2* overexpression increases cell invasion in vitro. Simultaneous knockdown of *WNT11* by RNAi could reverse these pro-metastatic effects of *ROR2* overexpression [149]. In several CRC cell lines, depletion of *WNT11* by RNAi or treatment with WNT11-targeting antibodies reduced their invasion capability. Upon overexpression of *WNT11* in CRC cell lines, an increased activity of an ATF2 reporter, which can be stimulated by JNK and p38-MAPK signaling, and a reduction of a Wnt reporter was observed. However, no mechanistic link between modulation of pathways and the invasive phenotype could be demonstrated in these models [74]. Similarly, overexpression of *WNT11* in the CRC cell line HCT116 activated Jun/JNK signaling and increased the invasion and migration ability of cells, with no effects on canonical Wnt signaling [77]. In prostate cancer cell lines, WNT11 was shown to signal via FZD8 and the transcription factor ATF2 to increase cell migration and invasion in Matrigel assays and 3D cell cultures [75]. In mouse breast cancer cell lines, knockdown of *Wnt11* reduced cell motility and protrusive activity. Mechanistically, it could be shown that Wnt11 is loaded onto fibroblast-derived exosomes by breast cancer cells and is then secreted in an autocrine manner to activate Wnt/PCP signaling via Fzd6 [150]. In the CRC cell lines RKO and SW480, exogenous addition of WNT3A and WNT5A could increase intracellular Ca^2+^ levels and increase cell migration in scratch assays, an effect which could be reverted by addition of a Phospholipase C (PLC) inhibitor [53]. These results indicate that both WNTs can increase CRC cell migration via Wnt/Ca^2+^-PLC signaling, despite WNT3A being known as an activator of canonical Wnt signaling (Table 1). Similarly, in SW480 cells, knockdown of *WNT5A* was shown to decrease directed cell migration, an effect which could be rescued by the addition of WNT5A-conditioned medium. The same study showed that in Apc1638N mice, a transgenic mouse model of CRC, overexpression of *WNT5A* had no effect on tumor size and expression of proliferation markers as assessed by immunohistochemistry. These observations suggest that WNT5A does not contribute to the initiation and progression of tumors driven by *APC* mutation. Further, no difference in expression of EMT markers in immunohistochemistry could be shown in this study [104]. Thus, it remains unknown whether the pro-metastatic effects of WNT5A are confined to in vitro models in CRC.

Beside pro-metastatic effects of WNTs secreted from the tumor cells themselves, WNTs secreted from the tumor microenvironment contribute to metastatic phenotypes. In a mouse model of breast cancer, invasion-promoting tumor-associated macrophages (TAM) were selected by their ability to co-migrate into a needle inserted into the tumor. Comparison of these invasion-promoting TAM with a bulk TAM population from the same mice revealed elevated expression of *WNT5B* and *WNT7B* in invasion-promoting TAMs. Yet, the precise contribution of those WNTs remains unknown [151]. Similarly, another study showed that TAMs in breast cancer biopsies express WNT5A. Upon co-culture with TAMs, the invasiveness of the breast cancer cell line MCF-7 in a Boyden chamber assay was drastically increased, an effect which could be emulated by addition of recombinant WNT5A. In this model, increased invasiveness was shown to be dependent on JNK/AP-1 signaling. Furthermore, WNT5A induced the expression of *MMP7* in MCF-7 cells in a JNK-dependent manner, possibly contributing to invasion by increasing extracellular proteolysis [152].

In summary, secreted WNTs from both tumor cells and adjacent cells from the TME play a role in various metastasis-associated subprocesses by both canonical and non-canonical Wnt signaling. Particularly the non-canonical ligands WNT5A/B and WNT11 strongly contribute to metastatic phenotypes by facilitating remodeling of the cytoskeleton and cell–cell-contacts via Wnt/PCP and Wnt/Ca^2+^ signaling. In addition, WNTs enhance metastasis by reshaping the extracellular matrix by matrix metalloproteases.

## 5. Secreted WNTs and Cancer Immune Evasion

**Figure 4:**
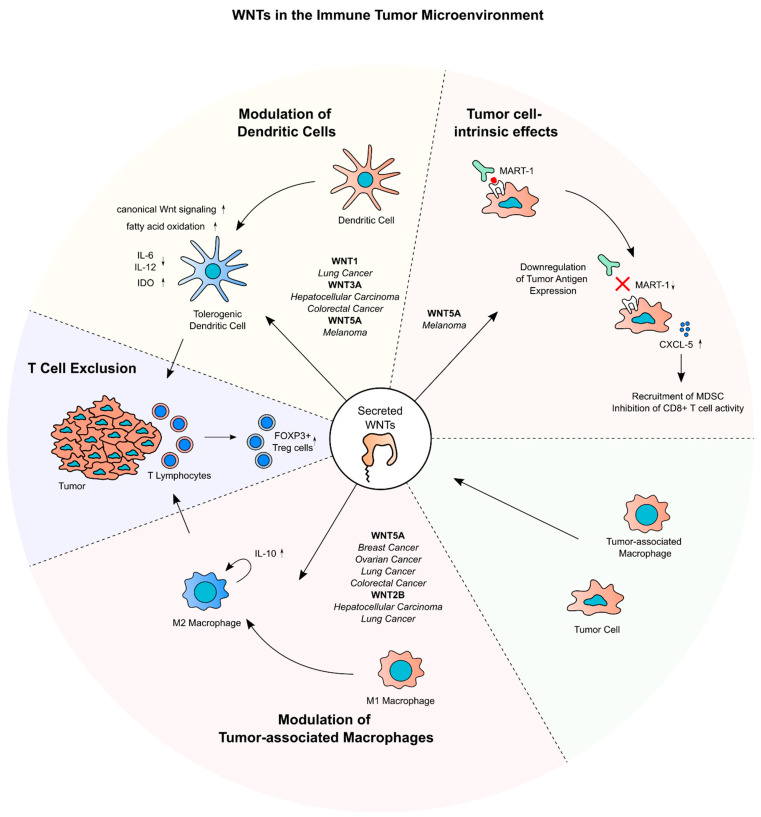
**Function of secretion of WNTs in tumor immune evasion.** Key processes in immune evasion and WNTs acting on them are shown. The cancer entities in which the processes have been observed are listed in italic. WNTs, especially WNT5A, contribute to the anti-inflammatory modulation of dendritic cells and macrophages in the tumor immune microenvironment. Both processes contribute to the exclusion of T cells from the microenvironment, for instance, by downregulation of specific chemokines. In melanoma, WNT5A downregulates the expression of MART-1, a tumor antigen, and alters chemokine expression in tumor cells themselves, contributing to a lowered activity of tumor-infiltrating T cells. Clinically, inhibition of WNT secretion by ETC-159 has shown to synergize with immune checkpoint inhibition in preclinical studies and pilot clinical trials.

In the recent years, the importance of the crosstalk between tumor cells and components of the innate and adaptive immune response for carcinogenesis and tumor progression has been widely recognized [153]. Therapeutically, these discoveries have enabled the development of immune checkpoint inhibitors and adoptive T-cell transfer [127,128,154]. Novel immunotherapeutic approaches have been proven clinically effective in subsets of cancer patients [155], but immune evasion mechanisms, such as immune cell exclusion or downregulation of tumor antigens, pose obstacles for their widespread application [128]. Here, we discuss the implication of Wnt signaling and especially secreted WNTs for immune homeostasis in cancer (Figure 4).

Several cell types are involved in anti-tumor-immunity. Dendritic cells (DC), T lymphocytes, as well as TAMs present in the immune TME, can be polarized to exhibit either tumor-promoting or tumor-suppressing effects [156,157]. For instance, TAMs can be polarized into M1 and M2 lineages. The M1 subtype is linked to inflammation, while the M2 subtype is associated with an anti-inflammatory phenotype [158,159]. In this context, cytokines secreted from tumor and stroma cells, such as IL-10, as well as chemokines like CCL4, play a crucial role in shaping the immune TME in a tumor-promoting manner [153]. In non-cancer tissues, Wnt signaling was shown to influence hematopoietic lineage commitment, immune cell differentiation, and cytokine secretion [39,160,161,162,163,164,165]. WNT5A, for instance, was shown to induce a regulatory phenotype in DCs by induction of IL-10 through Wnt/Ca^2+^ signaling [166]. These physiological mechanisms are often hijacked by cancers to drive immune escape.

### 5.1. WNTs and Tumor-Associated Dendritic Cells and T Cells

Active Wnt signaling in tumor cells and/or immune cells in the TME has been shown to favor the immune escape of tumor cells. For instance, knockdown of *WNT5A* in melanoma xenografts in mice resulted in a in a lower number of Foxp3+ regulatory T cells (Treg) in the tumors as well as in tumor-draining lymph nodes. In in vitro experiments, this effect could be attributed to a specific upregulation of Indole-2,3-dioxygenase (IDO) in DCs by WNT5A. These WNT5A-conditioned DCs then mediate the Treg polarization of naïve CD4+ T cells [167]. Additionally, DC-specific knockout of β-catenin in a melanoma mouse model of diminished IDO expression and augmented the antitumoral T-cell response, suggesting a WNT5A-β-catenin-IDO axis in DCs that promotes Treg polarization in melanoma. Beside this mechanism, WNT5A was shown to contribute to immune tolerance by inhibiting pro-inflammatory cytokines and inducing metabolic changes in DCs [168]. Concordantly, in a BRAF^V600E^ PTEN^–/–^ melanoma mouse model and a Kras^LSL-G12D/+^ Trp53^fl/fl^ non-small cell lung cancer mouse model, blockage of WNT secretion by ETC-159 or antagonization of WNTs by the anti-FZD-antibody Vantictumab led to an increase of intratumoral CD8+ T cells [169]. In lung cancer tissues, *WNT1* expression showed an inverse correlation with the proportion of tumor infiltrating cytotoxic T lymphocytes (CTLs). Knockdown of *WNT1* in a lung cancer xenograft mouse model led to an increased proportion of antigen specific CTLs in a lung cancer xenograft mouse model. Mechanistically, this could be attributed to downregulation of chemokines, such as CCL4 and CCL7 upon WNT1 overexpression in DCs. Confirming these results, administration of siWNT1 nanoparticles in a mouse model of chemically induced lung cancer led to reduced levels of nuclear β-Catenin in intratumoral DCs, increased CTL infiltration and a decrease of total tumor burden [44]. A similar observation was made in HCC and CRC mouse models, where addition of recombinant WNT3A to tumor-derived T cells increased expression of β-Catenin and decreased expression of the CTL differentiation marker T-bet. In this CRC mouse model, intratumoral administration of a WNT3A targeting antibody (aWNT3A) decreased tumor volume and an increased the proportion of tumor antigen-specific CTLs in the TME, which exhibited a higher expression of T-bet and IFN-γ. These results indicate an important role of WNT3A for repressing functionality of tumor-infiltrating CTLs [54,170]. This WNT3A-dependent polarization of T cells seems to be mostly dependent on DCs, since CTLs which were not exposed to aWNT3A still showed increased effector functions in aWNT3A CRC xenografts [54]. The studies above provide compelling evidence that specific WNTs contribute to T-cell exclusion from tumors by inducing canonical Wnt signaling in DCs.

Not only canonical Wnt signaling is implicated in driving polarization of T cells and their exclusion from the tumor. In a BRAF^V600E^ PTEN^–/–^ melanoma mouse model, expression of the non-canonical WNT5A was upregulated upon immune checkpoint inhibition with a PD-1-targeting antibody. In this model, WNT5A was shown to increase the expression of *CXCL5*, which then reduced the infiltration of myeloid-derived suppressor cells. Since co-occurring with WNT5A upregulation, a stabilization of YAP could be observed, indicating that the effects of WNT5A on chemokine expression in this model are mediated by Hippo signaling [60].

Apart from escaping the immune system by modulating adjacent immune cells, cancer cells often downregulate tumor-associated antigens to diminish the adaptive immune response towards the tumor [171]. In a subset of melanoma tissues with low expression of tumor antigens such as *MART-1*, the expression of WNT5A is increased. Consistently, overexpression of *WNT5A* in melanoma cell lines downregulated the expression of *MART-1*, which was shown to be dependent on a PKC-STAT3 signaling axis. Finally, in co-culture assays, treatment with recombinant WNT5A was shown to dampen the reactivity of CTL to melanoma cells [172].

### 5.2. WNTs and Tumor-Associated Macrophages

WNTs are also implied in modulating tumor-associated macrophages in the TME. For instance, in a xenograft mouse model of HCC, abrogation of the WNT secretion of tumor cells by knockdown of *WLS* decreased the proportion of tumor-infiltrating TAMs and Treg cells and increased the proportion of CD4+ and CD8+ T cells. This coincided with a reduction of tumor weight of the xenografts in this model [173]. Furthermore, Liu et al. could show that in CRC tissues, WNT5A expression correlated with the expression of the M2 polarization marker CD163 in macrophages. Upon co-culture with CRC cell lines, the macrophage cell line THP-1 cells upregulated *WNT5A* and subsequently *CD163* expression, which was dependent on a CaMKII-ERK-STAT3 signaling axis. By addition of an IL-10-targeting antibody, this effect could be reverted. Knockdown of *WNT5A* in these TAM reduced tumor formation in xenograft experiment with CRC cells. These results suggest that WNT5A supports CRC progression by promoting M2 polarization of TAMs in an autocrine manner through induction of IL-10 and Wnt/Ca^2+^ signaling [58].

Supporting these results, in tissue microarrays of breast cancer samples, a correlation between *WNT5A* expression in tumor cells and the percentage of M2 macrophages could be shown [165]. In HCC, *WNT2B* was shown to be expressed in M2 TAMs. Overexpression of *WNT2B* in THP-1 cells increased the expression of CD163. In TAMs derived from coculture with HCC cells, knockdown of *WNT2B* or β-catenin reduced the expression of CD163, indicating an effect of WNT2B-induced canonical Wnt signaling on M2 polarization of TAMs in HCC [51]. Corroborating these results, the expression of *WNT5A* and *WNT2B* correlated with the abundance of M2 macrophages in SCLC tissues [174]. Furthermore, in a xenograft *WNT5A*-knockout mouse model of ovarian cancer, several chemokines, among those CCL1 and CXCL10, were downregulated in the peritoneal lavage. This was accompanied by an elevation of CD8+-T cells and M1 TAM, as well a decrease of FOXP3+-Treg cells and M2 TAM in the tumor tissues, implying that WNT5A modulates the immune response to the tumor in a tolerogenic fashion [175].

In summary, several studies showed that WNTs derived from both tumor and stroma cells contribute to main mechanisms of cancer immune escape. First, different WNTs have been shown to prime DCs in a tolerogenic manner, which influences cytokine expression and leads to exclusion or tolerogenic polarization of tumor-infiltrating T cells. Secondly, secreted WNTs, especially WNT5A, were reported to be involved in M2 polarization of TAMs, leading to an altered cytokine profile and immune cell exclusion from the tumor.

## 6. Therapeutical Targeting of WNTs in Cancer

The various important functions of secreted WNTs for cancer biology renders WNTs attractive pharmacological targets for antineoplastic therapy. Several approaches to target WNTs in cancer have been investigated so far. Firstly, we provide an overview of the different pharmacological strategies to target secreted WNTs in cancer. Secondly, we summarize clinical trials that include therapeutics targeting WNTs in the treatment of cancer (Table 2). It is important to mention that so far, these trials remain restricted to phase I and II. Hence, these studies have not been designed to assess the clinical efficacy of the tested drugs in the treatment of the respective cancer entities, and the conclusions that can be drawn from them are limited.

### 6.1. Blockade of WNT Secretion

Small molecules inhibiting Porcupine, e.g., LGK-974, block general secretion of WNTs and are the most intensively studied antineoplastic drugs that target the WNT pathway. This class of drugs proved to effectively inhibit canonical Wnt signaling and to induce cancer cell death and slow tumor growth in preclinical animal models [120,188,189,190,191,192]. For instance, in HNSCC xenograft models, LGK-974 treatment strongly reduced Wnt target gene expression and tumor volume, in the absence of obvious adverse effects [190]. In patient-derived xenografts of *RSPO*-translocation CRC, the porcupine inhibitor ETC-159 could effectively diminish Wnt reporter activity and tumor volume [191]. In a MMTV-WNT1 breast cancer mouse model, Wnt-C59 treatment could reduce tumor growth [192]. Beside its effect on canonical Wnt signaling and cell proliferation, blockade of WNT secretion was shown to synergize with immune checkpoint inhibition due to the immunomodulatory effects of specific WNTs [169,193]. Several porcupine inhibitors entered early clinical trials, namely, LGK-974, ETC-159, RXC-004, and CGX-1321 [194,195,196,197]. In a phase 1 trial conducted in patients with various solid tumors, LGK-974 efficiently decreased Wnt target gene expression in normal and tumor tissues of most patients [177]. In another phase 1 trial including patients with several advanced solid tumors, ETC-159 was shown to inhibit Wnt signaling in tumor and normal tissues at tolerated doses [179]. The most observed adverse effects of porcupine inhibitors in these two studies were dysgeusia and loss of bone density. In both studies, no patient showed a response to PORCN inhibitor therapy in terms of tumor reduction [177,179], and their clinical potential and tumor entities to be targeted remain an open question.

In line with WNTs contributing to cancer immune escape in vitro, studies indicate that targeting WNT secretion could facilitate cancer immune therapy. In melanoma patients, it was shown that in tissue specimens of patients not responding to checkpoint inhibitors, expression of several WNTs was strongly upregulated [169]. Accordingly, in preclinical mouse studies, a synergism of WNT blockade and checkpoint inhibition could be shown in melanoma, lung cancer, and CRC models [169,193]. Furthermore, in a phase 1 trial including patients with a broad spectrum of solid tumors, the response of canonical Wnt signaling in tumor tissues to inhibition of WNT secretion by LGK-974 was associated with an increase in chemokine expression in tumor biopsies, indicating that targeting WNT secretion in solid tumors may have beneficial effects on the cytokine composition in the TME and the immune response towards the tumor [177].

### 6.2. Disruption of WNT Ligand–Receptor Interactions

Another strategy to diminish the effects of secreted WNTs on cancer cells is to intercept the interaction of WNTs with their cognate FZD receptors with anti-FZD antibodies. The anti-FZD antibody Vantictumab was evaluated for clinical use in metastatic pancreatic adenocarcinoma and metastatic HER2-negative breast cancer in phase I clinical trials [180,181]. Response to Vantictumab as determined by lower expression of Wnt target genes in the tumor tissue correlated with better overall survival, but the conclusions to be drawn are limited due to a low number of participants and a missing control arm [180]. In another study, Vantictumab treatment resulted in loss of bone density in some patients, whereas no conclusion about the anti-tumor efficacy could be drawn [181]. Furthermore, a decoy WNT-receptor, Ipafricept, was tested in clinical trials [182,184]. Overall, a combination of Ipafricept with paclitaxel and carboplatin in ovarian cancer patients showed a favorable response, but the interpretation of clinical efficacy is limited due to the design of the study. Overall, WNT- or FZD-targeting antibodies were well tolerated, but in some patients, they exhibited remarkable adverse effects on bone metabolism, such as pathological fractures [180,181,182,184].

Beside FZDs, ROR1 is utilized as a therapeutical target. In CLL, WNT5A-ROR1 signaling is an integral contributor to proliferation. Anti-ROR1-antibodies, for instance Cirmtuzumab have entered phase I trials in lymphoma [186,187]. In a cohort of 26 CLL patients, most patients achieved stable disease, measured by the absolute lymphocyte count during treatment with Cirmtuzumab. Furthermore, Cirmtuzumab reduced the expression of genes related to cancer stem cells, as well as Rac1 and HS1 signaling in CLL cells [186]. In various lymphoid cancers, Zilovertamab Vedotin, an anti-ROR1 antibody coupled to the microtubule inhibitor Auristatin E, could achieve partial response in the subgroup of patients with mantle cell lymphoma and diffuse large cell B-cell lymphoma [187].

Although targeting secreted WNTs in cancer by inhibiting WNT secretion or intercepting the interaction of WNTs with receptors seems to be a promising strategy for cancer treatment in preclinical trials, compelling evidence for their activity as single agent in early clinical phase trials is still lacking. Notably, since canonical Wnt signaling takes a pivotal role in adult tissue maintenance [198], specific adverse effects of the tested inhibitors and antibodies on bone metabolism could be observed [176]. The potential adverse effects of general Wnt inhibition could be overcome by targeting specific WNTs, e.g., through RNA interference or antibody-based therapeutics. In different mouse models of lung cancer, for instance, an efficient silencing of WNT1 in tumors through administration of siWNT1 nanoparticles could be demonstrated, which coincided with a lower tumor burden [44]. Furthermore, due to the pleiotropic nature of WNTs, treatments targeting secreted WNTs could be combined with drugs targeting other oncogenic pathways in cancer-specific manner. In preclinical studies, LGK-974 was proven to facilitate immune-checkpoint inhibition [60,193]. Combination therapy of PORCN and immune checkpoint inhibitors is currently under investigation in clinical trials, but no results have been published (NCT02675946, NCT04907539, and NCT02521844) [178]. As another example, in xenograft models of different WNT-dependent cancer entities, treatment with ETC-159 efficiently reduced the expression of DNA repair genes, rendering the cells susceptible to PARP inhibition [199]. Further, in xenograft mouse models of Wnt-addicted cancers, inhibition of Wnt secretion synergized with inhibition of mTOR/PI3K signaling to reduce tumor growth [189]. Together, these results suggest that general WNT inhibition alone may not suffice to effectively treat cancers, but that targeting of specific WNTs or combination of WNT inhibition with other therapeutical strategies may be a promising option.

## 7. Outlook

Over the last decades, many insights into the roles of WNTs in tumors have been obtained, revealing a spectrum of distinct functions during many steps of carcinogenesis, from tumor initiation to proliferation and metastasis. These diverse oncogenic phenotypes are not only driven by the effect of WNTs on canonical and non-canonical Wnt signaling, but also through its cross-talk with other oncogenic pathways. Furthermore, WNTs were shown to be important mediators of communication between cancers and cells in the tumor microenvironment. Paracrine signaling, mediated for instance by WNT5A, is important for tumor immune evasion, which can be observed across many different cancer entities. Despite these insights, many questions about the biological function of WNTs in cancer remain open. For instance, the WNT family comprises a large set of genes. While the functions of singular WNTs, such as WNT5A and WNT3, have been extensively described in cancer, other family members still need to be characterized in detail. In this respect, the distinct expression pattern of WNT family members in different tumor types suggest that there are tumor tissue- and stage-specific functions of WNTs for cancer biology. Thus, the biological function of all WNTs needs to be determined within specific, relevant contexts. Insights from this research will likely address controversies regarding the tumor-suppressive or -promoting function of specific WNTs, such as WNT5A.

Furthermore, in most cancers, several WNTs are expressed simultaneously, but their contribution to the net effect on tumor phenotypes is unclear. Hence, the contribution of single WNT family members and their interaction with other expressed WNTs needs to be disentangled. This is particularly important for cancer therapy, as general approaches to block WNT secretion remained challenge and might cause adverse effects. Identifying specific WNT family members drives cancer phenotypes in specific tumor entities, or learn how targeting WNT in combination with other targeted or immune therapies. Furthermore, compared to other pathways, there is still a limited number of pharmacological agents against WNT pathway components, and expanding this repertoire will hopefully turn the Wnt pathway into a druggable target in the future.

## Figures and Tables

**Table 1:** Effects of WNTs on canonical and non-canonical Wnt signaling in cancer; + activating effect reported, - inhibiting effect reported, * no effects reported.

Gene	Canonical Wnt Signaling	Wnt/ PCP	Wnt/Ca^2+^	Other Pathways	Reference
WNT1	+	*	*	EGFR-ERK1/2	[43,44,45,46]
WNT2	+	*	*		[47,48,49,50]
WNT2B	+	*	*		[51]
WNT3	+	*	*		[52]
WNT3A	+	*	+		[52,53,54]
WNT4	+	+	*		[55,56]
WNT5A	+/-	+	*	YAP/TAZ, p53 stabilization, MAPK-ERK, MAPK-Rac1-p38	[53,57,58,59,60,61,62,63]
WNT5B		+	*	YAP/TAZ, cell cycle, LAT1, MMP-10	[64,65,66]
WNT6	+	*	*		[67]
WNT7A	+	*	*	MMP-10	[68]
WNT7B	+	*	+		[69,70]
WNT8A	*	*	*		
WNT8B	+	*	*		[71]
WNT9A	*	*	*		
WNT9B	*	*	*		
WNT10A	*	*	*		
WNT10B	+	*	*	Wnt/STOP	[35,72]
WNT11	-	+	*	MAPK-p38	[73,74,75,76,77]
WNT16	+	*	*		[78]

**Table 2:** Overview of drugs targeting secreted WNTs investigated in clinical trials in cancer. A detailed review of Wnt secretion inhibitors and Fzd antagonists in cancer treatment was provided by Jung et al. [176].

Drug Class	Drug	Tumor Entity	Combination	Clinical Trial	Phase	Reference
PORCN inhibitor	LGK974	Various WNT dependent cancer		NCT01351103		Rodon et a. 2021 [177]
PORCN inhibitor	LGK974	BRAF-mutant metastatic colorectal cancer (mCRC)	LGX818 (PI3Ki) Cetuximab	NCT02278133	I/II	N/A
PORCN inhibitor	RXC004	(RNF43) or R-spondin (RSPO) aberrated, metastatic, microsatellite stable, colorectal cancer	Nivolumab Denosumab	NCT04907539	II	Kopetz et al. 2022 [178]
PORCN inhibitor	RXC004	Advanced malignancies		NCT03447470	I	Cook et al. 2021
PORCN inhibitor	RXC004	Advanced solid tumors	Denosumab	NCT04907851	II	N/A
PORCN inhibitor	CGX1321	Advanced GI Tumors	Pembrolizumab Encorafenib (Raf inhibitor) for BRAFV600E positive tumors	NCT02675946	I	N/A
PORCN inhibitor	XNW7201	Advanced solid tumors		NCT03901950	I	N/A
PORCN inhibitor	ETC159	Advanced solid tumors	Pembrolizumab	NCT02521844	I	Ng et al. 2017 [179]
FZD mAb	Vantictumab	metastatic HER2-negative breast cancer		NCT01973309	Ib	Diamond et al. 2020 [180]
FZD mAb	Vantictumab	Previously untreated stage IV pancreatic cancer	Nab-Paclitaxel Gemcitabine	NCT02005315	I	Davis et al. 2020 [181]
FZD mAb	Vantictumab	Previously treated NSCLC	Docetaxel	NCT01957007	I	N/A
Decoy WNT receptor	Ipafricept	Hepatocellular cancer		NCT02069145	I	N/A
Decoy WNT receptor	Ipafricept	Solid tumors		NCT01608867	I	Jimeno et al. 2017 [182]
Decoy WNT receptor	Ipafricept	Stage IV pancreatic cancer		NCT02050178	I	Dotan et al. 2019 [183]
Decoy WNT receptor	Ipafricept	recurrent platinum-sensitive ovarian cancer		NCT02092363	I	Moore et al. 2019 [184]
radiolabeled FZD10 mAb	OTSA101-DTPA	Synovial sarcoma		NCT04176016	I	N/A
radiolabeled FZD10 mAb	OTSA101-DTPA	Synovial sarcoma		NCT01469975		Giraudet et al. 2018 [185]
ROR1 mAb	Cirmtuzumab	Chronic lymphocytic leukemia		NCT02222688		Choi et al. 2018 [186]
ROR1-mAb-drug-conjugate	Zilovertamab vedotin	Lymphoid cancers		NCT03833180		Wang et al. 2021 [187]
